# Impact of Steroid Pulse Therapy on Early Treatment Response and Relapse in Type 1 Autoimmune Pancreatitis

**DOI:** 10.1002/jgh3.70448

**Published:** 2026-07-20

**Authors:** Akihisa Adachi, Itaru Naitoh, Michihiro Yoshida, Yasuki Hori, Akihisa Kato, Kenta Kachi, Tadashi Toyohara, Kayoko Oda, Yusuke Kito, Kenji Urakabe, Toshitaka Mori, Hiromi Kataoka

**Affiliations:** ^1^ Department of Gastroenterology and Metabolism Nagoya City University Graduate School of Medical Sciences Nagoya Japan; ^2^ Department of Gastroenterology Nagoya City University Midori Municipal Hospital Nagoya Japan

**Keywords:** adverse event, autoimmune pancreatitis, relapse, steroid pulse

## Abstract

**Aims:**

Despite the recommendation of steroid therapy for type 1 autoimmune pancreatitis (AIP), the value of steroid pulse therapy remains unclear. This study aimed to assess the efficacy and safety of steroid pulse therapy in patients with AIP.

**Methods and Results:**

Of 121 patients with type 1 AIP who were identified, 116 with adequate follow‐up data were included in the analysis. Patients were classified into four groups according to the initial treatment modality—pulse therapy alone (Pulse alone group), pulse therapy followed by oral steroid therapy (Pulse + PSL group), conventional oral steroid therapy (PSL group), and observation without treatment (Observation group)—and short‐ and long‐term clinical response, relapse rates, and steroid‐associated adverse events were compared across these groups. The numbers of patients in the Pulse alone, Pulse + PSL, PSL, and Observation groups were 12, 13, 68, and 23, respectively. After 2 weeks of steroid initiation, steroid pulse groups (Pulse alone and Pulse + PSL group) showed a significantly greater reduction in serum IgG4 levels than the PSL group (*p* = 0.048). Pulse + PSL was associated with a significantly lower relapse rate than PSL (log‐rank test *p* = 0.02; multivariate Cox regression *p* = 0.048), with no differences in adverse events. Rates of relapse and adverse events did not differ significantly between the Pulse alone and Observation groups.

**Conclusions:**

Steroid pulse therapy may serve as a useful therapeutic option when conducting a steroid trial and may help reduce relapse rate in the management of type 1 AIP.

## 1 Introduction

Autoimmune pancreatitis (AIP) is an immune‐mediated pancreatic inflammatory disease first described in 1995 [1], and was classified as types 1 and 2. Hereafter, AIP refers to type 1 AIP unless otherwise specified. AIP shows abundant IgG4‐positive plasma cells, storiform fibrosis, and obliterative phlebitis. AIP is considered a part of IgG4‐related disease (IgG4‐RD), and is characterized by elevated serum IgG4 levels. AIP is frequently accompanied by involvement of multiple organs, in the form of sclerosing cholangitis, dacryoadenitis, sialadenitis, retroperitoneal fibrosis, and nephritis, all of which show similar pathological findings (other organ involvement; OOI) [2, 3, 4].

AIP is characterized by a marked response to steroid therapy. Hence, Japanese and International Consensus Diagnostic Criteria (ICDC) for AIP have incorporated effectiveness of steroid therapy as a diagnostic item [5]. Symptoms such as objective jaundice, pain, and symptomatic extrapancreatic lesions are indications for steroid therapy [2]. While oral prednisolone (PSL) is recommended for the induction of remission and as maintenance therapy, steroid pulse therapy has also been reported as an initial steroid treatment. However, the efficacy and safety of steroid pulse therapy have not been fully elucidated to date.

A key clinical issue in AIP is the lack of a highly accurate diagnostic biomarker. In particular, the differential diagnosis between AIP and pancreatic cancer (PC) is sometimes difficult, although serum IgG4 and histological examination by endoscopic ultrasound‐guided tissue acquisition (EUS‐TA) are useful. Elevated serum IgG4 levels are commonly observed, and they are considered a useful diagnostic marker for AIP. A meta‐analysis revealed a sensitivity and specificity of elevated serum IgG4 levels in the differential diagnosis of AIP of 0.72 and 0.93, respectively [6]. However, since elevated serum IgG4 levels are sometimes observed in PC, it is difficult to distinguish AIP from PC based only on serum IgG4 levels [5]. The response rate to steroid therapy in patients with AIP was reported to be 98.6% [7]. Therefore, steroid responsiveness is regarded as the diagnostic marker with the greatest sensitivity and specificity for differentiating between AIP and PC [8]. Several studies have evaluated treatment responsiveness of AIP using a steroid trial with oral PSL, assessing radiological or clinical improvement after 2 weeks [8, 9]. However, only a few reports have applied a similar response‐assessment approach using steroid pulse therapy alone.

In cases in which patients are ultimately diagnosed with PC after an oral PSL trial, discontinuing steroid therapy can be challenging, particularly when surgery or chemotherapy is planned. In this context, employing steroid pulse therapy for diagnostic purposes may mitigate this limitation. In addition, in cases where patients exhibit no pancreatic symptoms suggestive of AIP but demonstrate IgG4‐related sclerosing cholangitis as OOI, distinguishing this condition from cholangiocarcinoma can be particularly difficult. In such situations, steroid pulse therapy may offer diagnostic value.

AIP patients with no symptoms do not require steroid treatment. Since approximately 16% of AIP patients were managed without steroid therapy in a Japanese nationwide study [7], observation without steroid therapy is often chosen for asymptomatic individuals to avoid the potential adverse events (AEs) of steroids. In such cases, employing steroid pulse therapy to assess steroid responsiveness may allow for a shorter induction period and help prevent unnecessary continuation of steroid treatment.

Another clinical concern is the occurrence of relapse. Low dose maintenance steroid therapy is reported as being beneficial in lowering the risk of relapse [10, 11, 12]. Additionally, several studies have reported the use of pulse regimens as an initial steroid treatment for AIP, primarily because this approach can induce a marked improvement in associated biliary strictures [13, 14, 15]. Since the clinical challenges in AIP include the prolonged duration of steroid therapy recommended in current guidelines [2], which raises concerns about AEs, Ikeura et al. suggested that steroid pulse‐alone regimens (mPSL; 125–500 mg/day) may represent a potential therapeutic alternative for AIP, particularly in terms of minimizing steroid‐related AEs [16]. In addition, Sugimoto et al. reported that steroid pulse therapy (mPSL; 125 or 250 mg/day) was associated with improved relapse‐free survival in patients with diffuse pancreatic swelling [17]. Thus, steroid pulse therapy may be useful as remission induction therapy; however, the optimal dosing regimen has not been established. Based on these considerations, the present study evaluated the efficacy of steroid pulse therapy in patients with AIP.

## 2 Methods

### 2.1 Study Design

This was a single‐center, retrospective study conducted at Nagoya City University Graduate School of Medical Sciences. We retrospectively analyzed the records of patients who were diagnosed with type 1 AIP from September 1998 to September 2025 at our hospital. Patient's characteristics, demographics, laboratory data, treatment, history of relapse, and AEs were evaluated. This study was approved by the Institutional Review Board of Nagoya City University Hospital (approval No. 60‐25‐0139) and was conducted in accordance with the principles of the Declaration of Helsinki.

### 2.2 Patients and Treatment

A total of 121 patients were diagnosed as definitive or probable type 1 AIP according to the ICDC [5]. The day of initiation of steroid therapy was designated as Day 1, and based on previously published reports [2, 14, 18], each treatment regimen was defined as follows: Pulse alone group: Methylprednisolone, 500 mg/day, was administered on Days 1–3 and 8–10; Pulse + PSL group: Following completion of the methylprednisolone pulse, as in the Pulse alone group, 20 mg/day of oral PSL was initiated on Day 15 and tapered thereafter, with a maintenance dose of 2.5–5 mg/day; PSL group (Conventional treatment group): Oral PSL at 0.6 mg/kg/day was initiated on day 1 and administered for 2–4 weeks before being tapered and subsequently maintained at 2.5–5 mg/day; Observation group: Observation without steroid therapy. The steroid dosage and treatment schedule of steroid pulse therapy is shown in Figure S1. Although the treatment strategy was generally determined at the discretion of the attending physicians, steroid pulse therapy (Pulse alone and Pulse + PSL) was actively used from 2009 to 2013. Thereafter, treatment was generally administered in accordance with clinical guidelines, and steroid pulse therapy was essentially employed as a steroid trial. In principle, after tapering off the initial PSL dose, maintenance therapy was subsequently administered for approximately 3 years, after which steroid treatment was withdrawn; however, some patients continued treatment at the discretion of the attending physician.

### 2.3 Definitions

Parenchymal imaging was performed using computed tomography (CT) or magnetic resonance cholangiopancreatography (MRCP), and classified according to the ICDC criteria [5]. OOI related to AIP was also defined in accordance with the ICDC criteria as the presence of characteristic abnormalities in the proximal bile duct (biliary lesions), sialadenitis, retroperitoneal fibrosis, or kidney lesions. Imaging changes following treatment were evaluated using CT or MRCP. The degree of improvement in pancreatic thickness was assessed by measuring the transverse diameter of the pancreas along the anterior aspect of the vertebral body in cases with diffuse pancreatic enlargement, whereas the transverse diameter of the affected segment was measured in cases with focal enlargement. Relapse was defined as the reappearance of symptoms attributable to IgG4‐RD that required either re‐initiation or dose escalation of steroid therapy. For patients in the Observation group, relapse was characterized by the need for administration of steroid therapy at any point during the follow‐up period. Data on steroid‐associated AEs, including osteoporosis and fractures, infection, glucose metabolic disturbances, cardiovascular events, and ophthalmologic disease, were systematically collected. AEs were assessed from treatment initiation to the end of follow‐up. In this study, the effects of each treatment type were evaluated separately as short‐term and long‐term outcomes. “Short‐term” was defined as the period around 14 days from the initiation of steroid therapy, whereas “long‐term” was defined as the period extending until the end of clinical follow‐up. For the short‐term analysis, the primary endpoint was the effectiveness of steroid therapy, as assessed by changes in serum IgG4 levels and pancreatic thickness, while the primary endpoint for long‐term analysis was relapse. The secondary endpoint, common to both analyzes, was the occurrence of AEs.

### 2.4 Comparison of Treatment Modalities

The short‐term therapeutic efficacy of steroid pulse therapy was evaluated by comparing the Pulse alone and Pulse + PSL groups with the PSL group. For this efficacy analysis, we used blood data obtained approximately 14 days after the initiation of steroid therapy. The long‐term therapeutic effects in the Pulse + PSL and PSL groups were assessed based on the occurrence of relapse. In addition, long‐term disease progression in patients undergoing pulse therapy alone and observation alone was examined. In the Observation group, the time to the event was calculated from the date of diagnosis.

### 2.5 Statistical Analysis

All statistical analyzes were performed using EZR software. Categorical variables were examined using Fisher's exact test, while continuous variables were evaluated using the Mann–Whitney U test. Time‐to‐event outcomes were analyzed using the Kaplan–Meier curves and the log‐rank test. Risk factors for relapse were evaluated using Cox univariate and multivariate analyzes. Parameters with a *p*‐value < 0.20 in univariate analyzes were included in multivariate analysis. Values of *p* < 0.05 were considered significant.

## 3 Results

### 3.1 Study Flowchart

**FIGURE 1:**
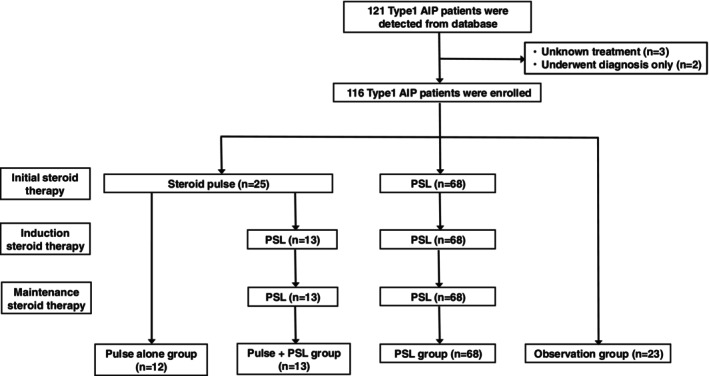
Flowchart of the inclusion of patients with autoimmune pancreatitis in this study. AIP, autoimmune pancreatitis; PSL, prednisolone.

A total of 121 patients were identified from the database. Among them, five patients were excluded (three had an unknown treatment modality and two had been diagnosed at our institution but received treatment elsewhere), and 116 patients were included in this study. One hundred three patients were diagnosed as definitive AIP, and 13 as probable AIP based on ICDC [5]. A flowchart of the study is presented in Figure 1. Twelve patients were treated with steroid pulse therapy alone (Pulse alone group), 13 patients received oral PSL maintenance therapy following steroid pulse therapy (Pulse + PSL group), 68 patients were treated with oral PSL alone (PSL group), and 23 patients were followed up without steroid therapy (Observation group).

### 3.2 Clinical Profiles of the Patients

Patients' characteristics at diagnosis are shown in Table S1. The median age of the entire cohort was 69 years, and the male: female ratio was 91:25 (78% vs. 22%). Median serum levels of IgG, IgG4, and HbA1c were 1740 (725–5041) mg/dL, 332 (2–2960) mg/dL, and 6.2% (4.6% – 13.1%), respectively. Regarding parenchymal imaging, 48 (41%) patients demonstrated diffuse type AIP. OOI was identified in 60 (52%) patients. This included biliary lesions in 30 patients, and sialadenitis, retroperitoneal fibrosis, and renal lesions in 20 patients each. The median follow‐up period was 2390 days. The characteristics of patients in each treatment group are shown in Table 1.

**TABLE 1:** Patient characteristics of each treatment group.

		Pulse alone group (*N* = 12)	Pulse + PSL group (*N* = 13)	PSL group (*N* = 68)	Observation group (*N* = 23)
Age, median (range)		69.5 (55–79)	70 (52–81)	67.5 (43–87)	75 (56–83)
Sex (male), n		10 (83%)	11 (85%)	54 (79%)	16 (70%)
Serum IgG, median (range)	(mg/dL)	1612 (1017–3012)	1851 (1211–3241)	1771 (930–4931)	1493 (721–5041)
Serum IgG4, median (range)	(mg/dL)	205 (35.8–540)	489 (55.6–1550)	396 (2–2620)	209 (27.7–2960)
Serum total bilirubin, median (range)	(mg/dL)	0.55 (0.1–7.6)	0.6 (0.3–5.4)	1.65 (0.3–24.7)	0.6 (0.2–15.3)
Serum amylase, median (range)	(U/L)	67 (38–195)	77 (33–345)	91 (14–924)	90 (28–559)
Serum HbA1c, median (range)	(%)	6.3 (5.2–9.5)	5.9 (5.0–11.3)	6.2 (4.6–13.8)	6.2 (5.3–8.6)
Parenchymal imaging, *n*	Diffuse	1 (8%)	8 (62%)	34 (50%)	5 (22%)
Number of patients with OOI	Total	2 (17%)	6 (46%)	46 (68%)	6 (26%)
	Biliary lesions, *n*	1 (8%)	3 (23%)	23 (34%)	3 (13%)
	Sialadenitis, *n*	0 (0%)	2 (15%)	16 (24%)	2 (9%)
	Retroperitoneal fibrosis, *n*	1 (8%)	2 (15%)	15 (22%)	2 (9%)
	Renal lesions, *n*	0 (0%)	3 (23%)	15 (22%)	2 (9%)

Abbreviation: OOI: Other organ involvement.

### 3.3 Comparison of Short‐Term Outcomes Between Steroid Pulse and Conventional Treatment (“Pulse Alone and Pulse + PSL Group” vs. “PSL Group”)

First, we compared the Pulse alone and Pulse + PSL group (Pulse group) with the PSL group to assess short‐term outcomes. Comparison of the characteristics of patients is shown in Table S2. There were no significant differences in age, sex, IgG levels, or IgG4 levels at baseline between the two groups. Total bilirubin and the number of OOI lesions were significantly higher in the PSL group. Table 2 demonstrates the outcomes assessed 2 weeks after steroid administration. Steroid effectiveness was observed in all 25 patients who received steroid pulse therapy. There was no significant difference between the two groups in the interval from blood sampling to treatment initiation. Serum IgG and IgG4 levels, and the reduction in pancreatic thickness did not differ between the two groups. However, the decrease in serum IgG4 levels was significantly greater in the Pulse group (*p* = 0.048). No AEs were observed over the short‐term period in either group. Glycemic control did not differ between the two groups.

**TABLE 2:** Comparison of outcomes 2 weeks after steroid initiation between “Pulse group” and “PSL group.”

		Pulse group* (*n* = 25)	PSL group (*n* = 68)	*p*
Days from steroid administration (range)		14 (13–28)	14 (10–33)	0.75
Serum IgG, median (range)	(mg/dL)	1078 (713–1853)	1259 (194–3028)	0.12
Serum IgG4, median (range)	(mg/dL)	209 (15.8–726)	260 (13.5–1750)	0.34
Serum HbA1c, median (range)	(%)	6.6 (5.5–8.1)	6.5 (5.4–10.9)	0.60
Post/pre serum IgG (range)	(%)	63 (45–85)	74 (13–112)	0.07
Post/pre serum IgG4 (range)	(%)	61 (37–87)	67 (15–114)	0.048
Pancreatic thickness rate (range)	(%)	56 (37–119)	69 (35–122)	0.15
Addition of antidiabetic agents, *n*		8 (32%)	19 (28%)	0.80
Adverse events, *n*		0 (0%)	0 (0%)	1.00

*Note:* Post/pre serum IgG: serum IgG (day14 / baseline), Post/pre serum IgG4: serum IgG4 (day14/baseline), Pulse group*: Pulse alone group and Pulse + PSL group.

### 3.4 Long‐Term Outcomes of Steroid Pulse Therapy as the Initial Steroid Therapy Compared With Conventional Therapy (“Pulse + PSL Group” vs. “PSL Group”)

**FIGURE 2:**
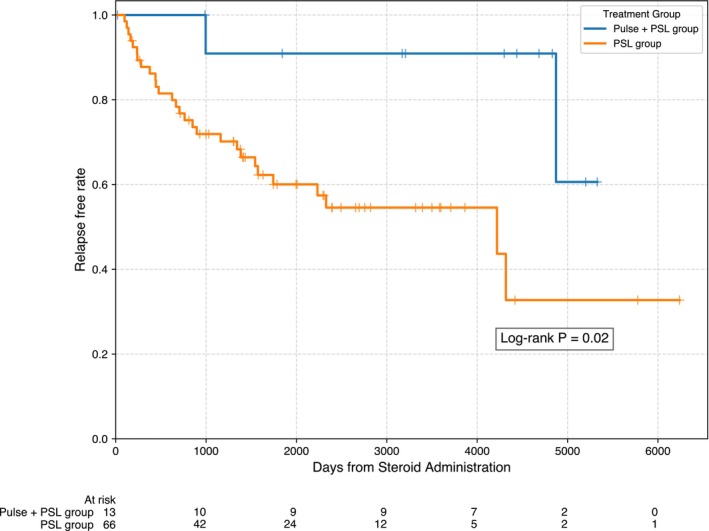
Kaplan–Meier curves for relapse‐free survival in the Pulse + PSL group and the PSL group.

There were no significant differences in the baseline characteristics of patients in the Pulse + PSL group and PSL group (Table S3). Kaplan–Meier analysis demonstrated that Pulse + PSL group patients had a significantly lower relapse rate than those in the PSL group (Figure 2, log‐rank test, *p* = 0.02). In univariate analysis, PSL group (*p* = 0.03) and total bilirubin level at baseline (*p* = 0.04) were significantly associated with relapse. In multivariate analysis, PSL group was the only independent predictor of relapse (hazard ratio, 4.56; 95% CI 1.01–20.05; *p* = 0.048) (Table 3). There was no significant difference in the cumulative incidence of AEs between the PSL group and the Pulse + PSL group (Table 4 and Figure S2, log‐rank test, *p* = 0.92).

**TABLE 3:** Univariate and multivariate analyzes of relapse‐associated factors in autoimmune pancreatitis: “Pulse + PSL” versus “PSL group.”

		Univariate analysis			Multivariate analysis		
		HR	95% CI	*p*	HR	95% CI	*p*
Treatment (PSL group)		4.92	1.13–21.41	0.03	4.56	1.01–20.50	0.048
Sex (male)		0.45	0.14–1.50	0.19	0.62	0.18–2.13	0.44
Age		1.01	0.97–1.04	0.76			
Serum IgG, median	(mg/dL)	1	1.00–1.00	0.42			
Serum IgG4, median	(mg/dL)	1	1.00–1.00	0.91			
Serum total bilirubin, median	(mg/dL)	1.07	1.00–1.13	0.04	1.04	0.97–1.11	0.25
Serum amylase, median	(U/L)	1	1.00–1.00	0.93			
Serum HbA1c, median	(%)	0.94	0.74–1.19	0.62			
OOI total		1.98	0.88–4.45	0.1	1.6	0.69–3.68	0.27
Biliary lesions		1.25	0.59–2.64	0.56			
Sialadenitis		1.12	0.48–2.62	0.79			
Retroperitoneal fibrosis		0.98	0.40–2.41	0.97			
Renal lesions		1.35	0.58–3.19	0.49			
Parenchymal imaging		1.49	0.72–3.07	0.28			
Capsule–like rim		0.6	0.29–1.24	0.17	0.63	0.30–1.31	0.21
Calcification		0.45	0.06–3.34	0.43			

Abbreviation: OOI: Other organ involvement.

**TABLE 4:** Adverse events in each treatment group.

	Pulse alone group (*N* = 12)	Pulse + PSL group (*N* = 13)	PSL group (*N* = 68)	Observation group (*N* = 23)
Addition of antidiabetic agents	2 (17%)	4 (31%)	16 (24%)	5 (22%)
Osteoporosis and fractures	0 (0%)	0 (0%)	6 (8.9%)	0 (0%)
Infection	1 (8.3%)	3 (23.1%)	6 (8.9%)	0 (0%)
Cardiovascular events	2 (16.6%)	0 (0%)	3 (4.4%)	1 (4.3%)
Ophthalmologic diseases	0 (0%)	1 (7.7%)	1 (1.5%)	0 (0%)
Others	0 (0%)	0 (0%)	1 (1.5%)	0 (0%)
Total	3 (25%)	4 (30.8%)	17 (25%)	6 (27%)

*Note:* Others: adrenal insufficiency.

### 3.5 Long‐Term Outcomes Between Steroid Pulse Monotherapy and Observation Alone (“Pulse Alone Group” vs. “Observation Group”)

**FIGURE 3:**
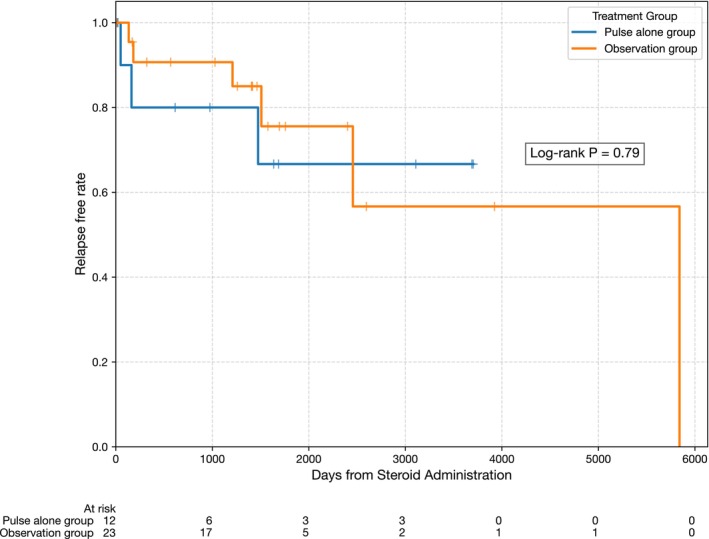
Kaplan–Meier curves for relapse‐free survival in the Pulse alone group and the Observation group. In the Observation group, the time to the event was calculated from the date of diagnosis.

Comparison of the baseline characteristics between patients in the Pulse alone and Observation groups is presented in Table S4. Relapse rates did not differ significantly between the two groups (Figure 3, log‐rank test, *p* = 0.79). Additionally, as shown in Table 4, there were no significant differences in the rate of AEs between the two groups.

## 4 Discussion

In this study, we investigated the potential utility of steroid pulse therapy in the management of AIP. The results showed that the short‐term response to treatment was comparable between steroid pulse therapy and conventional steroid therapy, and a potential advantage of steroid pulse therapy was suggested. Furthermore, the relapse rate was significantly lower with steroid pulse therapy compared with conventional steroid therapy when used as the initial steroid therapy.

The steroid trial is an important diagnostic‐cum‐therapeutic intervention for distinguishing AIP from PC. Reportedly, the diagnostic confirmation of AIP requires EUS‐TA, a steroid trial, or surgical resection [19]. One case report described the usefulness of steroid pulse therapy in distinguishing AIP from PC [15]. Moon et al. reported a high diagnostic performance of steroid therapy for differentiating AIP from PC in a prospective outcome study [8]. In the present study, although the degree of improvement in pancreatic enlargement did not differ between steroid pulse and conventional oral steroid therapy, the pre‐ to posttreatment IgG4 ratio was significantly more favorable in the steroid pulse treatment group in the early treatment phase. Therefore, steroid pulse therapy may be an effective approach for assessing the therapeutic responsiveness of AIP.

One of the key clinical challenges during the treatment and follow‐up of AIP is the risk of relapse. The relapse rate of AIP has been reported to be approximately 30% [20]. For the treatment of AIP, initiation of steroid therapy with 0.6 mg/kg/day PSL, followed by tapering of the dose and subsequent maintenance therapy is recommended [2, 17]. In contrast, reports supporting the efficacy of the steroid pulse therapy for AIP remain limited. However, steroid pulse therapy may have potential benefits in terms of the long‐term outcomes of AIP. Ikeura et al. reported that steroid pulse therapy (mPSL; 125–500 mg/day) might be a beneficial alternative to oral steroid therapy as the initial therapy for the induction of remission in patients with AIP; however, relapse rates were comparable between the pulse‐plus‐maintenance PSL group and the oral PSL alone group [16]. Sugimoto et al. demonstrated that, although steroid pulse therapy with mPSL at 125 or 250 mg/day was associated with improved relapse‐free survival in patients with AIP presenting with diffuse pancreatic parenchymal enlargement on imaging, there was no significant difference in the 5‐year relapse rate in the overall AIP cohort [17]. In terms of the manifestations of IgG4‐related disease, although treatment protocols differed, steroid pulse therapy has been reported to be more effective than oral PSL in patients with IgG4‐related ophthalmic disease [21]. In our analysis, we evaluated a protocol using mPSL at 500 mg/day. Unlike previous studies, our analysis showed that steroid pulse therapy as remission induction therapy was associated with a significantly lower relapse rate than conventional oral steroid therapy. These findings suggest that remission induction therapy using the present protocol may represent an effective therapeutic option. Separately, as an example suggesting the potential effectiveness of steroid pulse therapy, Ikeura et al. also reported a case in which a lower bile duct stricture that did not improve with oral steroid therapy improved after steroid pulse therapy [16]. Since biliary lesions represent an important disease condition that necessitates steroid therapy, the suppression of biliary lesion activity by steroid pulse therapy may have contributed to the favorable outcomes observed in our study.

There are multiple reports of AEs associated with steroid pulse therapy, particularly those involving cardiovascular complications [22, 23, 24]. According to a review summarizing severe complications associated with high‐dose intravenous methylprednisolone pulse (IVMP) therapy for Graves' orbitopathy, the risks of myocardial infarction, acute heart failure, hepatic failure, and cerebrovascular events warrant careful monitoring of cardiovascular risk and liver function during treatment, and the cumulative dose of IVMP is recommended not to exceed 8 g [22]. However, most of the previous reports evaluated IVMP administered at 1000 mg per day. Furthermore, a meta‐analysis comparing the safety of steroid pulse therapy with that of oral steroids has shown that pulse therapy does not increase the incidence of serious AEs [25]. In the present study, we evaluated a methylprednisolone regimen of 500 mg per day, and no difference in the cumulative incidence of complications compared with conventional therapy was observed. Taken together, these findings suggest that steroid pulse therapy may be equal to or superior to conventional oral steroid therapy as the initial treatment for the induction of remission in terms of relapse rates and AEs.

Some AIP patients are asymptomatic or experience spontaneous remission. In these cases, steroid therapy is not mandatory. In most patients who improve spontaneously, bile duct stenosis is absent and elevation of serum IgG4 levels is less common [26, 27]. These patients can be managed with observation without steroid therapy; however, distinguishing them from PC occasionally remains a clinical challenge. As noted above, the steroid trial is a useful option in this setting, although the clinical outcomes of steroid pulse alone have not been well established. In the present study, we demonstrated that long‐term outcomes did not differ significantly between patients who received steroid pulse therapy for the steroid trial and those managed by observation without steroid therapy. These results indicate that a steroid trial with steroid pulse therapy alone may be a promising approach as it allows assessment of steroid responsiveness without adversely affecting long‐term outcomes such as relapse and adverse events.

This study has several limitations. First, this was a single‐center retrospective study. Second, although the steroid regimens generally followed the guideline, some variations existed depending on the clinical judgment of the attending physicians. Third, patients in the conventional treatment group had higher total bilirubin levels and a greater number of OOIs, which may have biased the outcomes in favor of steroid pulse therapy. Fourth, the relatively small number of patients who received steroid pulse therapy and the limited number of relapse cases precluded a robust evaluation of the characteristics of patients who may benefit most from steroid pulse therapy. Although the results of the multivariate analysis in our study suggest that steroid pulse therapy may be more effective than conventional oral PSL therapy in preventing relapse, prospective multicenter studies are warranted to validate these findings.

Importantly, with the availability of inebilizumab as maintenance therapy for preventing relapse in IgG4‐RD, effective steroid‐based remission induction therapy is becoming increasingly important [28]. Compared with conventional oral PSL therapy, steroid pulse therapy may be useful as a means of assessing steroid responsiveness, including in the differential diagnosis from pancreatic cancer. In addition, based on the lower relapse rate observed in our study, steroid pulse therapy may be expected to serve as a more potent steroid‐based remission induction therapy than oral PSL therapy.

In conclusion, steroid pulse therapy showed no safety concerns in AIP and may be a promising alternative to oral steroid therapy for diagnostic steroid trials and initial remission induction.

## Funding

The publication of this article was supprted by JSPS KAKENHI Grant Number JP25K11258.

## Conflicts of Interest

The authors declare no conflicts of interest.

## Supporting information


**Table S1:** Patient characteristics at diagnosis.
**Table S2:** Patient characteristics of “Pulse group” and “PSL group.”
**Table S3:** Patient characteristics of “Pulse + PSL group” and “PSL group.”
**Table S4:** Patient characteristics of “Pulse alone group” and “Observation group.”

**Figure S1:** Flowchart of the treatment schedule mPSL; methylprednisolone, PSL; prednisolone.

**Figure S2:** Kaplan–Meier curves for adverse event‐free survival in the Pulse + PSL group and the PSL group.

## Data Availability

The data that support the findings of this study are available from the corresponding author upon reasonable request.
