# Supplementary material for: Impact of Steroid Pulse Therapy on Early Treatment Response and Relapse in Type 1 Autoimmune Pancreatitis
Source: JGH Open. 2026 Jul 20;10(7):e70448. doi: 10.1002/jgh3.70448 (PMC13384910; doi:10.1002/jgh3.70448)
Supplement: Supplementary file 1 — Table S1: Patient characteristics at diagnosis. Table S2: Patient characteristics of “Pulse group” and “PSL group.” Table S3: Patient characteristics of “Pulse + PSL group” and “PSL group.” Table S4: Patient characteristics of “Pulse alone group” and “Observation group.” [file JGH3-10-e70448-s002.docx]

**Supplementary Table 1. Patient characteristics at diagnosis**

|  |  | **(N=116)** |
| --- | --- | --- |
| Age, median (range) |  | 69 (43**–**87) |
| Sex (male), n |  | 91 (78.4%) |
| Serum IgG, median (range) | (mg/dL) | 1740 (725**–**5041) |
| Serum IgG4, median (range) | (mg/dL) | 332 (2**–**2960) |
| Serum total bilirubin, median (range) | (mg/dL) | 0.7 (0.1**–**24.7) |
| Serum amylase, median (range) | (U/L) | 83.5 (14**–**924) |
| Serum HbA1c, median (range) | (%) | 6.2 (4.6**–**13.8) |
| Parenchymal imaging, n | diffuse | 48 (41%) |
| Number of patients with OOI | Total | 60 (52%) |
|  | Biliary lesions, n | 30 (26%) |
|  | Sialadenitis, n | 20 (17%) |
|  | Retroperitoneal fibrosis, n | 20 (17%) |
|  | Renal lesions, n | 20 (17%) |

OOI: Other organ involvement

**Supplementary Table 2. Patient characteristics of “Pulse group” and “PSL group”**

|  |  | **Pulse group^*^**  **(n=25)** | **PSL group**  **(n=68)** | **p-value** |
| --- | --- | --- | --- | --- |
| Age, median (range) |  | 70 (52**–**81) | 67.5 (43**–**87) | 0.56 |
| Sex (male), n |  | 21 (84%) | 54 (79%) | 0.77 |
| Serum IgG, median (range) | (mg/dL) | 1758 (1017**–**3241) | 1771 (930**–**4941) | 0.62 |
| Serum IgG4, median (range) | (mg/dL) | 314 (35.8**–**1550) | 396 (2**–**2620) | 0.52 |
| Serum total bilirubin, median (range) | (mg/dL) | 0.6 (0.1**–**7.6) | 1.65 (0.3**–**24.7) | 0.01 |
| Serum amylase, median (range) | (U/L) | 69 (33**–**345) | 90.5 (14**–**924) | 0.21 |
| Serum HbA1c, median (range) | (%) | 6.1 (5**–**11.3) | 6.2 (4.6**–**13.8) | 0.28 |
| Parenchymal imaging, n | diffuse | 9 (36%) | 34 (50%) | 0.25 |
| Number of patients with OOI | Total | 8 (32%) | 46 (68%) | <0.01 |
|  | Biliary lesions, n | 4 (16%) | 23 (34%) | 0.12 |
|  | Sialadenitis, n | 2 (8%) | 16 (24%) | 0.14 |
|  | Retroperitoneal fibrosis, n | 3 (12%) | 15 (22%) | 0.38 |
|  | Renal lesions, n | 3 (12%) | 15 (22%) | 0.38 |

OOI: Other organ involvement, Pulse group^*^: Pulse alone group and Pulse + PSL group

**Supplementary Table 3. Patient characteristics of “Pulse + PSL group” and “PSL group”**

|  |  | **Pulse + PSL group (N=13)** | **PSL group**  **(N=68)** | **p-value** |
| --- | --- | --- | --- | --- |
| Age, median (range) |  | 70 (52**–**81) | 67.5 (43**–**87) | 0.67 |
| Sex (male), n |  | 11 (85%) | 54 (79%) | 1.00 |
| Serum IgG, median (range) | (mg/dL) | 1851 (1222**–**3241) | 1770.5 (930**–**4941) | 0.99 |
| Serum IgG4, median (range) | (mg/dL) | 489 (55.6**–**1550) | 396 (2**–**2620) | 0.29 |
| Serum total bilirubin, median (range) | (mg/dL) | 0.6 (0.3**–**5.4) | 1.65 (0.3**–**24.7) | 0.12 |
| Serum amylase, median (range) | (U/L) | 77 (33**–**345) | 90.5 (14**–**924) | 0.34 |
| Serum HbA1c, median (range) | (%) | 5.9 (5**–**11.3) | 6.2 (4.6**–**13.8) | 0.27 |
| Parenchymal imaging, n | diffuse | 8 (62%) | 34 (50%) | 0.55 |
| Number of patients with OOI | Total | 6 (46%) | 46 (68%) | 0.21 |
|  | Biliary lesions, n | 3 (23%) | 23 (34%) | 0.53 |
|  | Sialadenitis, n | 2 (15%) | 16 (24%) | 0.72 |
|  | Retroperitoneal fibrosis, n | 2 (15%) | 15 (22%) | 0.73 |
|  | Renal lesions, n | 3 (23%) | 15 (22%) | 1.00 |

OOI: Other organ involvement

**Supplementary Table 4. Patient characteristics of “Pulse alone group” and “Observation group”**

|  |  | **Pulse alone group (N=12)** | **Observation group (N=23)** | **p-value** |
| --- | --- | --- | --- | --- |
| Age, median (range) |  | 70 (55**–**79) | 75 (56**–**83) | 0.13 |
| Sex (male), n |  | 10 (83%) | 16 (70%) | 0.45 |
| Serum IgG, median (range) | (mg/dL) | 1612 (1017**–**3012) | 1493 (725**–**5041) | 0.70 |
| Serum IgG4, median (range) | (mg/dL) | 204.5 (35.8**–**540) | 209 (27.7**–**2960) | 0.39 |
| Serum total bilirubin, median (range) | (mg/dL) | 0.55 (0.1**–**7.6) | 0.6 (0.2**–**15.3) | 0.35 |
| Serum amylase, median (range) | (U/L) | 66.5 (38**–**195) | 90 (28**–**559) | 0.55 |
| Serum HbA1c, median (range) | (%) | 6.3 (5.2**–**9.5) | 6.2 (5.3**–**8.6) | 0.69 |
| Parenchymal imaging, n | diffuse | 1 (8.3%) | 5 (22%) | 0.64 |
| Number of patients with OOI | Total | 2 (17%) | 6 (26%) | 0.69 |
|  | Biliary lesions, n | 1 (8.3%) | 3 (13%) | 1.00 |
|  | Sialadenitis, n | 0 | 2 (8.7%) | 0.54 |
|  | Retroperitoneal fibrosis, n | 1 (8.3%) | 2 (8.7%) | 1.00 |
|  | Renal lesions, n | 0 | 2 (8.7%) | 0.54 |

OOI: Other organ involvement
