# Supplementary figures and images for: Impact of Steroid Pulse Therapy on Early Treatment Response and Relapse in Type 1 Autoimmune Pancreatitis
Source: JGH Open. 2026 Jul 20;10(7):e70448. doi: 10.1002/jgh3.70448 (PMC13384910; doi:10.1002/jgh3.70448)

## Pulse group

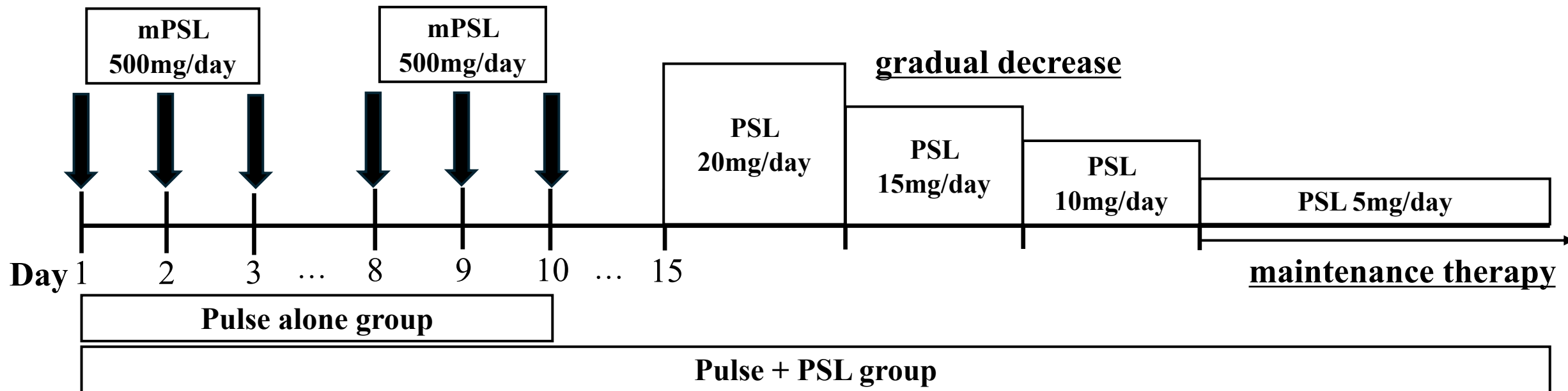

## PSL group

PSL 0.6 mg/kg day

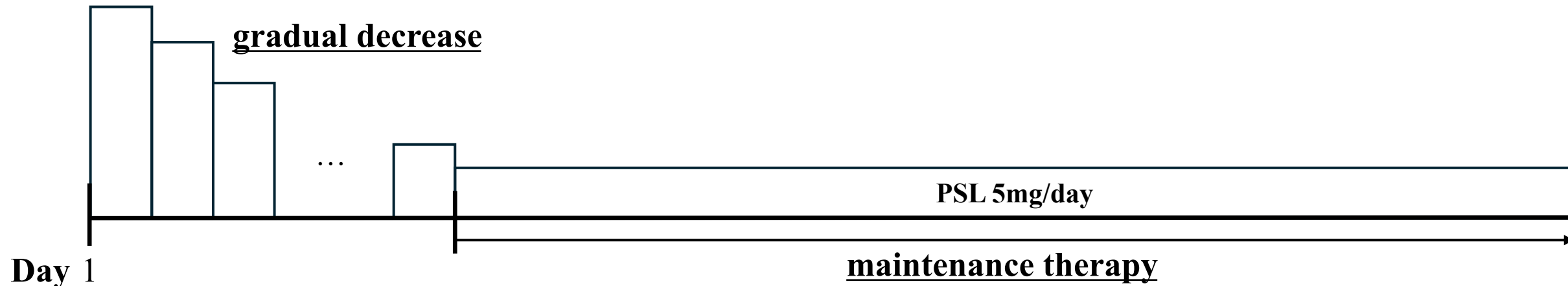

Supplement: Supplementary file 2 — Figure S1: Flowchart of the treatment schedule mPSL; methylprednisolone, PSL; prednisolone. [file JGH3-10-e70448-s001.pdf]

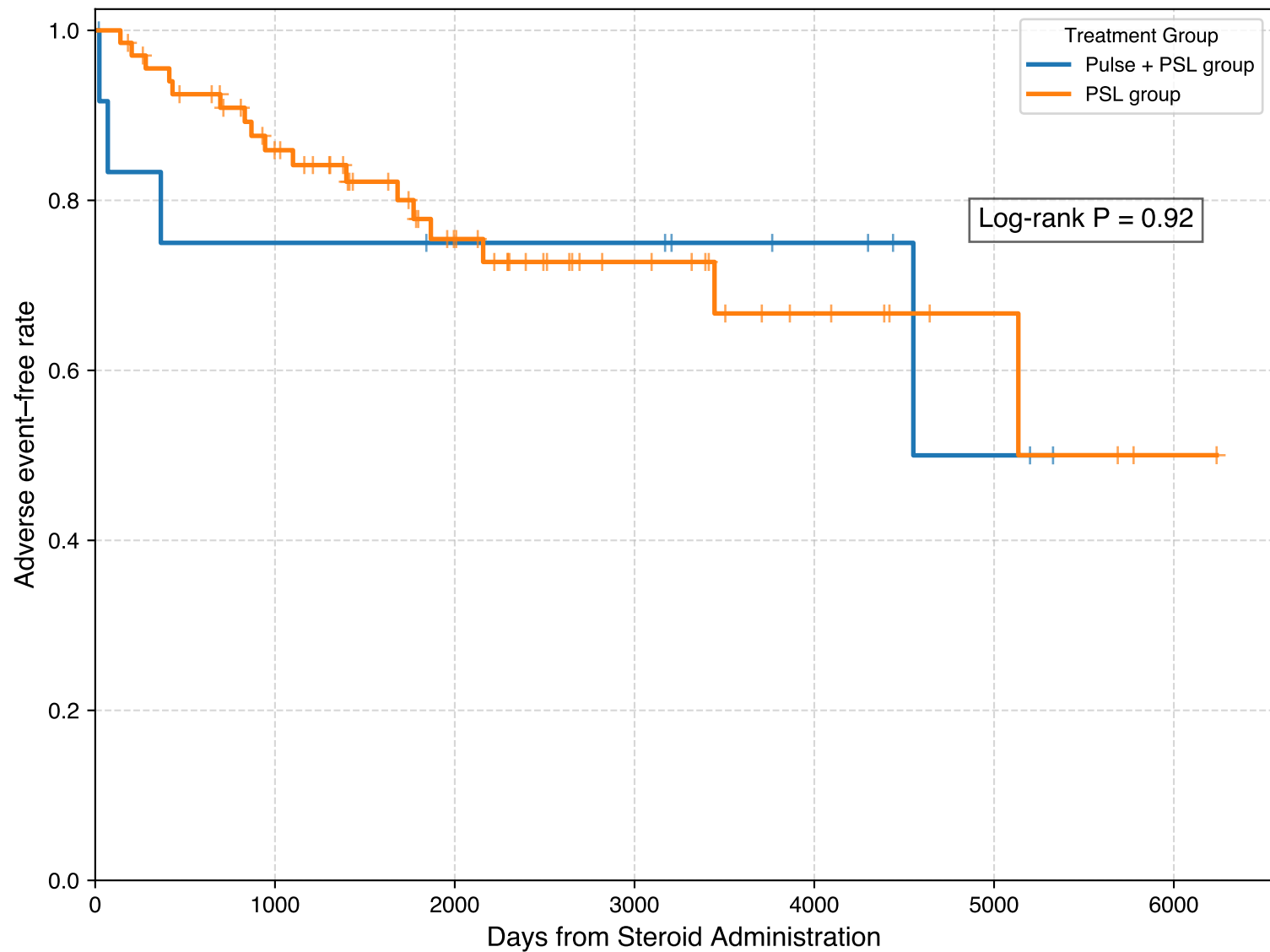

At risk

|                   |    |    |    |    |   |   |   |
|-------------------|----|----|----|----|---|---|---|
| Pulse + PSL group | 13 | 9  | 8  | 8  | 5 | 2 | 0 |
| PSL group         | 68 | 50 | 30 | 16 | 8 | 4 | 1 |

Supplement: Supplementary file 3 — Figure S2: Kaplan–Meier curves for adverse event‐free survival in the Pulse + PSL group and the PSL group. [file JGH3-10-e70448-s003.pdf]
